# Interleukin-37 expression and its potential role in oral leukoplakia and oral squamous cell carcinoma

**DOI:** 10.1038/srep26757

**Published:** 2016-05-26

**Authors:** Lin Lin, Jiayi Wang, Dongjuan Liu, Sai Liu, Hao Xu, Ning Ji, Min Zhou, Xin Zeng, Dunfang Zhang, Jing Li, Qianming Chen

**Affiliations:** 1 State Key Laboratory of Oral Diseases, West China Hospital of Stomatology, Sichuan University, Chengdu, Sichuan, China; 2 Nanjing Stomatological Hospital, Medical School of Nanjing University, Nanjing, Jiangsu, China

## Abstract

Interleukin 37 (IL-37) has been reported to play a significant role in innate immune response and to be involved in several kinds of cancers. However, the investigation of association between IL-37 and oral mucosa carcinogenesis hasn't been clearly established. The aim of the study was to assess IL-37 expression and explore its role in oral mucosa carcinogenesis. The expression of IL-37 increased from normal control (NC) to Oral leukoplakia (OLK) and oral squamous cell carcinoma (OSCC). Moreover, statistically highly significant difference was present between scores of OLK with and without mild/moderate dysplasia (P < 0.001). In addition, IL-37 expression was lower in OSCC with lymph node metastasis than those without metastasis (P < 0.01). What’s more, overexpression of IL-37 in RAW264.7 cells remarkably reduced the pseudopodia, vacuolization and the expression of IL-6, TNF-α, and IL-1β. Finally, we found IL-37 and its receptor IL-18Rα but not its binding partner IL-18BP have similar tissue location and expression trend in different stages of oral mucosa carcinogenesis. Overall, IL-37 can be used as a biomarker for early oral tumorigenesis and for malignant transformation risk assessment of premalignant lesions.

Carcinogenesis of the oral mucosa refers to the progressive development of oral squamous cell carcinoma from a normal mucosa, sometimes accompanied by metastasis^1^. It is a multi-stage process involving changes in the expression of a number of related genes. Oral leukoplakia is defined as “a white plaque of questionable risk having excluded (other) known diseases or disorders that carry no increased risk for cancer”. It is the best-known, potentially malignant disorder of the oral mucosa^2^. Histopathological grading of epithelial dysplasia is clinically one of the most important predictors of malignant potential^3^. OSCC is one of the most common malignancies worldwide and is characterized by low survival rate and high morbidity^4^,^5^. This is attributed to the lack of effective biomarkers for both early diagnosis and appropriate treatment^6^,^7^. A large number of genetic variants and signalling pathways have been associated with OSCC, but the contribution of these genes to the process of carcinogenesis remains unclear^8^. Therefore, there is an urgent need to develop novel and effective biomarkers for the improvement of OSCC prognosis.

The interleukin 1 (IL-1) family of ligands has 11 members and most of them are pro-inflammatory in nature. IL-1F7, also known as IL-37, is an important member of IL-1 family. There are five different splice variants (IL-37a–e) of IL-37, among them IL-37b is widely studied^9^,^10^. Expression of IL-37 mRNA is observed in a variety of tissues including testis, thymus, uterus, lymph nodes, bone marrow, placenta, and lungs^11^. IL-37 protein expression could be up-regulated by pro-inflammatory cytokines such as IL-1β, TNF-α, and IFN-γ in peripheral blood mononuclear cells (PBMCs) and in dendritic cells (DCs)^12^,^13^.

The function of IL-37 in inhibiting the innate immune response has been proven. It can activated by caspase-1 cleaves, then act as a cytokine through intracellular or extracellular pathways^14^. Both of the precursor and mature IL-37 could bind to IL-18Rα. Mature IL-37 also can combine with IL-18BP, the natural antagonist of IL-18, in an extracellular manner^15^,^16^. Earlier studies have demonstrated that IL-37 expression is closely associated with many diseases, such as inflammatory dermatosis, inflammatory hepatopathy, digestive system disorders, autoimmune diseases, metabolic diseases, I/R (ischemia/reperfusion) injury, and so on^17^,^18^,^19^,^20^,^21^,^22^,^23^. Inflammation is considered as the seventh hallmark of cancers^24^, suggesting that the anti-inflammatory properties of IL-37 might influence inflammation-related tumours. However, the function of IL-37 in the development of OSCC remains elusive. Based on the data described above, we hypothesized that IL-37 might be associated with the process of carcinogenesis in the oral mucosa by curbing pro-inflammatory cytokines. Here, we offer the first evidences that IL-37 can be used as a biomarker for early oral tumourgenesis and for risk assessment of malignant transformation in premalignant lesions.

## Results

### IL-37 expression increase from normal control to oral precancerous lesions and OSCC

**Figure 1:**
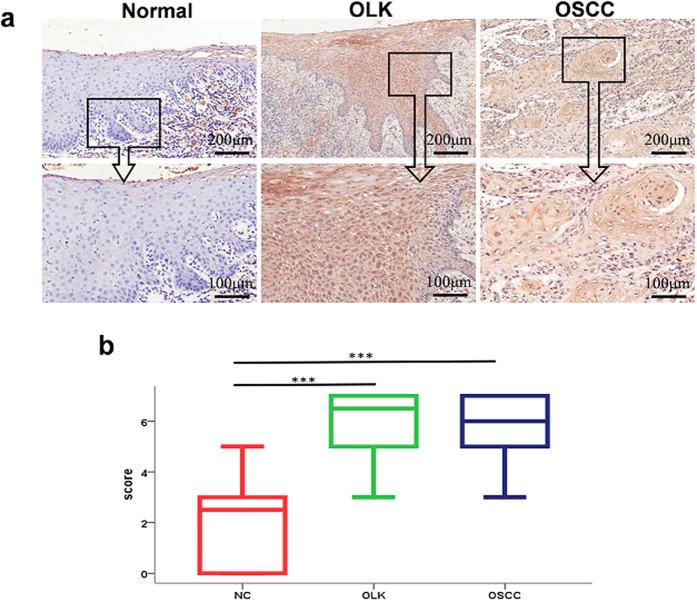
The expression of IL-37 protein in different stages of carcinogenesis tissues. (**a**) Immunohistochemical analysis of IL-37 expression in human healthy oral mucosa, OLK and OSCC lesions. (**b**) Comparison of the staining score between these groups (***P < 0.001).

IL-37 expression has been associated with many types of cancers, such as the stroma of colon carcinomas, gastric cardiac adenocarcinoma, and ductal mammary carcinoma^10^,^25^,^26^. However, the expression level and the function of IL-37 in the carcinogenesis of the oral mucosa are unknown. To investigate the expression of IL-37 in oral diseases, we performed immunohistochemistry on local-pathologically-changed tissues from individuals with OLK and OSCC. Diseased oral mucosa shows higher IL-37 expression than normal tissue, and according to analysis, the difference in IL-37 protein expression is statistically significant (n = 10, ***P < 0.001) (Fig. 1a,b). The clinical characteristics of these participants are listed in Table 1. These results suggest that the IL-37 protein expression is increased during the oral mucosa cancerous process.

### IL-37 expression is significantly difference between OLK patients with and without epithelial dysplasia

**Figure 2:**
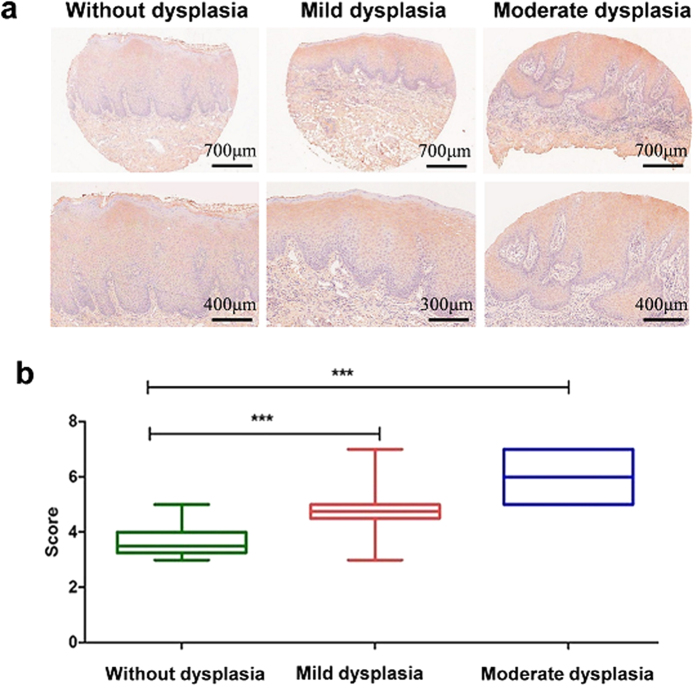
Relationship between IL-37 expression level and the degree of epithelial dysplasia in patients with OLK. **(a)** Immunohistochemical analysis of IL-37b expression in OLK lesions without epithelial dysplasia (top panels), with mild dysplasia (middle panels) and with moderate dysplasia (bottom panels). **(b)** Comparison of the staining score between these groups (***P < 0.001).

It has been demonstrated that diseased synovial lining contains large amounts of IL-37 protein, which is also true for the intestinal mucosa of active ulcerative colitis and in Crohn’s disease^11^,^18^. In addition, high expression of IL-37 was found in human psoriatic plaques and in atopic dermatitis skin^22^,^27^. OLK has attracted much attention due to its potential for malignant transformation where oral epithelial dysplasia is the most important prognostic indicator^28^. We analyzed the relationship between IL-37 levels and the degree of epithelial dysplasia in patients with OLK through a tissue microarray (TMA). Forty-five patients were recruited for this TMA, among them, twelve patients had mild epithelial dysplasia, four patients had moderate epithelial dysplasia, and twenty nine patients did not show any epithelial dysplasia. IL-37 immunoreactivity was detected clearly in OLK patients with both mild and moderate epithelial dysplasia, mainly in the keratin layer, spinous layer, and granular layer. IL-37 was also detected in patients without epithelial dysplasia, but was much weaker than the other two groups (Fig. 2a,b). The clinical characteristics of these OLK patients are listed in Table 2. However, no significant differences were found between the mild and moderate groups.

### IL-37 expression is lower in OSCC with lymph node metastasis than in those without metastasis

**Figure 3:**
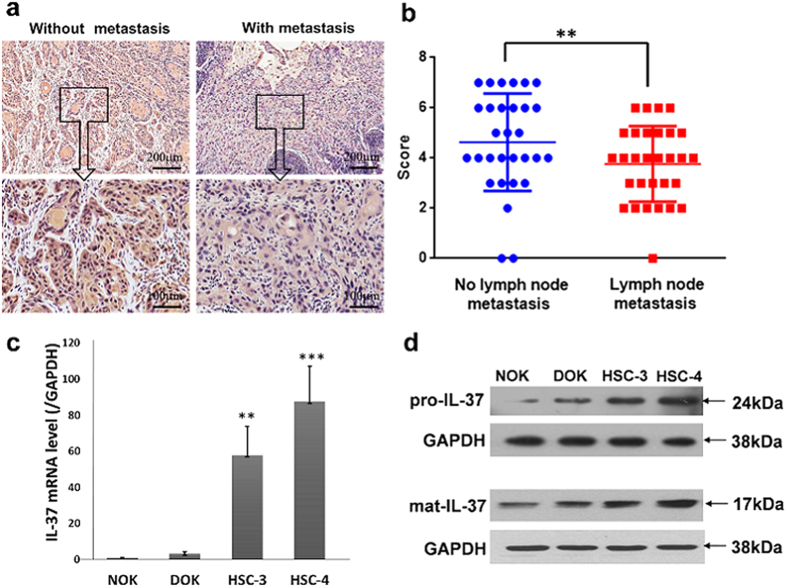
The expression of IL-37 in OSCC with or without metastasis ability. **(a)** Immunohistochemical analysis of IL-37b expression in OSCC patients with/without metastasis. **(b)** Comparison of the staining score between the two groups (*P = 0.004 < 0.01). (**c**) The expression of IL-37 mRNA in several cell lines detected by Q-PCR. (**d**) The expression of IL-37 protein (both pro-IL-37 and mat-IL-37) in several cell lines detected by Western blot.

OSCC has a high incidence of cervical micrometastasis and contralateral metastasis, because of the rich lymphatic intercommunication^29^. Thus, we hypothesised that there may be a correlation between IL-37 expression and OSCC metastasis. Immunohistochemistry was performed to investigate this hypothesis in OSCC patients with or without lymph node metastasis. As shown in Table 3, IL-37 expression is associated with lymph node status (P = 0.006), but had no association with the tumour size (P = 0.365). The distribution and average intensity of IL-37 staining are different between these two groups. The group without lymph node metastasis displayed strong IL-37 staining, mainly at the cytoplasmic region, and the group with metastasis displayed moderate IL-37 expression at the canter of carcinoma nest (Fig. 3a,b). Meanwhile, IL-37 expression is not associated with differentiation status (P = 0.350), smoking history (P = 0.636), gender, or age of the patients.

Next, we assessed the expression level of IL-37 in normal oral keratinocyte cells (NOK), dysplastic oral keratinocyte cells (DOK), and two human OSCC cell lines (HSC-3 and HSC-4). Our data showed that IL-37 mRNA and protein (both pro-IL-37 and mat-il-37) levels were obviously elevated in HSC-3 and HSC-4 cells, rather than in the NOK and DOK cells (HSC-4>HSC-3>DOK>NOK). Consistently, IL-37 mRNA and protein (both pro-IL-37 and mat-il-37) levels are all lower in the HSC-3 cells with stronger migratory potential than in HSC-4 cells (Fig. 3c,d and Supplementary Fig. 1). Collectively, these results suggest that IL-37 may contribute to the suppression of tumour metastasis.

### IL-37b abrogates the expression of pro-inflammatory cytokines in THP-1 cells

**Figure 4:**
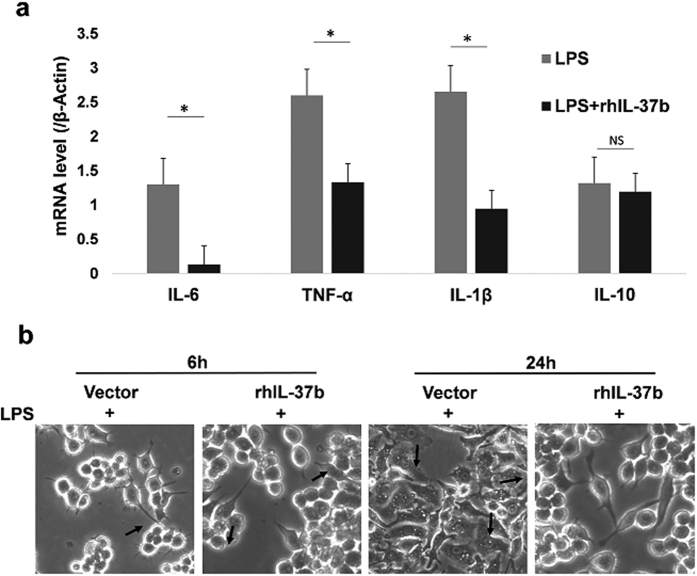
Cytokine production in THP-1 cells and the influence of polarization in RAW264.7 cells treated with the rhIL-37b. **(a)** The graph depicts that the gene transcript of IL-6, TNF-α and IL-1β of the LPS+rhIL-37b group was statistically significantly lower than the control group. There was no significant difference in the gene expression of IL-10. **(b)** IL-37b expression affects RAW264.7 cell polarization. RAW264.7 cells were detached from the flasks, plated into 6-well plates, pretreated with 10 ng/ml LPS and allowed to grow overnight, then treated with 1 μg/ml recombinant human IL-37b (right) or left untreated for control (left), photographed 6 h and 24 h later. The images are representative of 3 independently performed experiments.

IL-37 demonstrates an anti-tumour effect in fibrosarcoma^30^ and in HCC^31^, the mechanism has been determined using immune cells. The pro-inflammatory cytokines IL-6, TNF-α, and IL-1β have been reported to promote malignant transformation and tumour aggression in oral cancer^32^,^33^,^34^. We performed experiments using recombinant IL-37b to evaluate the effect of IL-37 on IL-6, TNF-α and IL-1β expression in THP-1 cells. In comparison with control cells, recombinant IL-37b treated cells showed decreased production of LPS-stimulated IL-6, TNF-α, and IL-1β, but not IL-10 (Fig. 4a). Moreover, RAW264.7 cells treated with IL-37b shows striking morphological differences, compared with the control cells, IL-37 treated RAW264.7 cells exhibited non-obvious or absence pseudopodia and vacuolization (Fig. 4b). These results suggest that IL-37b could change cell polarization through the down-regulation of pro-inflammatory cytokines in macrophages, which contributes to its anti-tumour effect.

### IL-37 and its receptor IL-18Rα have the same expression pattern

**Figure 5:**
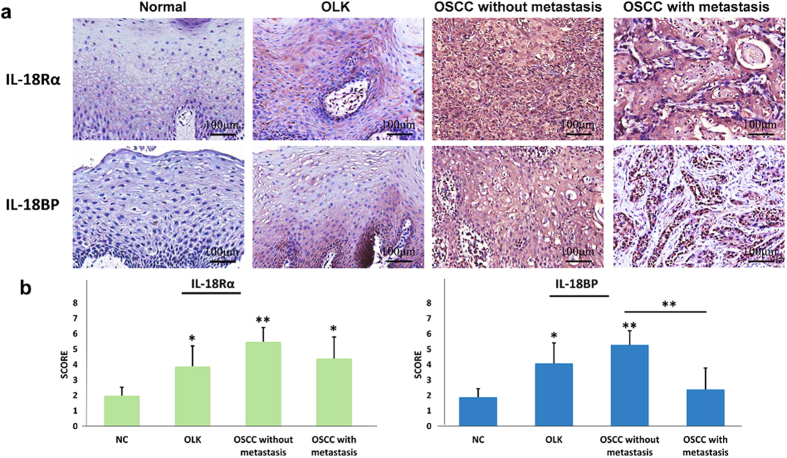
The expression level of the IL-37 receptor IL-18Rα and binding partner IL-18BP in NC, OLK and OSCC tissues. **(a)** Immunohistochemical analysis of IL-18Rα and IL-18BP expression in NC, OLK and OSCC tissues. **(b)** Comparison of the staining score between these groups (*P < 0.05, **P < 0.01).

To determine the activity of the IL-37 system, the expression level of its receptor IL-18Rα and its binding partner IL18BP were assessed. We enrolled 40 patients, including 20 cases of OSCC (10 patients with lymphatic metastasis and 10 patients without lymphatic metastasis), 10 cases of OLK and 10 cases of normal control from the same cohorts above used. IL-18Rα shows higher expression in OLK and OSCC tissues than normal control, but there is no difference between OSCC patients with and without lymphatic metastasis (Fig. 5a,b and Supplementary Table 2). Similarly, OLK and OSCC tissues without metastasis contained large amounts of IL-18BP, but there was no statistical difference between NC and OSCC metastasis (Fig. 5a,b and Supplementary Table 2). Overall, we could find IL-37 and its receptor IL-18Rα have similar tissues location and expression trend in different stages of oral mucosa carcinogenesis, but not its binding partner IL-18BP.

## Discussion

The mechanisms involved in IL-37-mediated inhibition of signalling pathways of the innate and acquired immune responses are poorly identified, due to its activities as a cytokine with both intracellular and extracellular functions^35^. In combination with IL-18Rα and the orphan receptor SIGIRR/IL-1R8, which is required as the signalling subunit, IL-37 could negatively regulate many signalling pathways, such as PTEN (phosphatase and tensin homolog deleted on chromosome ten) and STAT-3 (signal transducers and activators of transcription)^36^,^37^. Moreover, it can prevent the functional impairment of IL-7R signalling and the expression of purine synthesis genes in pro-B cell progenitors^38^.

IL-37 is a fundamental inhibitor of innate immunity that is mainly expressed by monocytes, macrophages, and epithelial cells^39^,^40^. Since inflammation has now been confirmed as the seventh hallmark of tumours, many speculate that the anti-inflammatory cytokine IL-37 might influence inflammation-related tumours^24^. Recently, IL-37 has been associated with several cancers, such as colon cancer, cardiac carcinoma, and breast cancer^10^,^25^,^26^. A previous study showed that a single injection of IL-37 could inhibit the growth of fibrosarcoma in mice, and that after multiple injections growth was completely inhibited^30^. Reduction of inflammation in aged mice via transgenic expression of IL-37 could prevent NRASV12-mediated oncogenesis^41^. Expression of IL-37 was decreased both in human hepatocellular carcinoma tissues and in renal cell carcinoma, suggesting that IL-37 may be useful for tumour immunotherapy^31^,^42^. The present study focused on the expression and regulatory role of IL-37 in the local environment of OLK and OSCC.

In the current study, IL-37 protein expression in the oral mucosa at different stages of oral carcinogenesis was analysed for the first time. Surprisingly, the expression pattern of IL-37 from normal oral mucosa to OSCC did not decrease, as observed in renal cancer^42^. Instead, there was only hardly detectable expression of IL-37 protein in all 10 samples of the normal oral mucosa, whereas staining was observed in some of the OLK and OSCC samples. Moreover, the expression of IL-37 in keratinocytes is region-specific, mainly located in keratin layer and spinous layer, similarly with pan-keratin expression pattern (Supplementary Fig. 2). Previous studies also indicated that the expression of IL-37 could be up-regulated by IL-1β, IL-10, and TNF-α^13^,^43^. Notably, this special expression pattern of IL-37 is very similar with human beta-defensin-1, which was recently reported by our group^44^. Thus, the most probable speculation is that the expression of this anti-inflammatory cytokine in OLK and OSCC may be a response of the body to inflammation. Further studies are being planned to prove this hypothesis.

OLK is at risk of malignant transformation and the discrimination between potentially malignant and non-harmful lesions^45^. Recently, OLK was proposed as a useful model for exploring the molecular mechanisms of oral carcinogenesis^46^. Our results showed that IL-37 is dramatically overexpressed in OLK, and to gain further insight into IL-37 expression at different degrees of epithelial dysplasia in OLK, TMA was used. Pathological evidence from our TMA indicated that OLK with epithelial dysplasia have positive IL-37b expression (P = 0.001). This is the first time that IL-37 has been reported as a potential predictor for malignant potential in OLK patients, especially in patients with epithelial dysplasia. It should be noted that the number of OLK patients with moderate/severe epithelial dysplasia in this study was limited. Despite this limitation, this study suggests IL-37 as a potentially novel diagnostic biomarker, which may be involved in the early steps of oral malignant transformation. Further studies are warranted to perfect this experiment. Previous studies have confirmed that IL-37 can suppress cell proliferation and invasion in cervical cancer^47^. Similarly, IL-37 was found negatively associated with OSCC tumour metastasis and cell motility in the present study. These observations indicate that IL-37 may play an important role in inhibiting OSCC metastasis.

The precise mechanism of how IL-37 regulates oral mucosa cancer is still unknown. IL-37b is known to be stably and constitutively expressed in macrophages and epithelial cells in small amounts, which can be increased dose dependently upon stimulation with LPS^11^. Our results showed that the effects of IL-37 are not only limited to morphological polarization, but also include decreased production of pro-inflammatory cytokines, including IL-6, TNF-α, and IL-1β, but not IL-10. Consistently, a previous study has shown that IL-37 expression in these kinds of cells almost completely suppressed the production of several major pro-inflammatory cytokines, such as IL-1α, IL-1β, IL-6, TNF, and MIP-2 *in vitro*^15^. Nevertheless, according to recently reports, IL-37 expression could be up-regulated by stimulation with IL-1β, IL-10, and TNF-α^13^,^43^. Taken together, these data revealed that IL-37 could act as a negative feedback inhibitor of inflammatory responses, and that this function does not depend on anti-inflammatory cytokines^48^, which could partly explain why IL-37 plays a role in the early steps of oral malignant transformation and contributes to the progress of oral carcinogenesis. Additionally, we found IL-37b treated RAW264.7 cells could obviously decrease pseudopodia formation and vacuolization. As we know, pseudopodia can extend toward higher concentrations of chemoattractants, which plays a key role in cell migration and cancer cell invasion^49^.

Since IL-37 expression increased from normal control to oral precancerous lesions and OSCC which we confirmed above, our results further showed that the expression of IL-37 and IL-18Rα are quite consistent. For example, both of IL-37 and IL-18Rα immunoreactivity was detected clearly at spinous layer in OLK tissues, and displayed strong staining in cytoplasmic region in OSCC tissues with/without metastasis. Unlike IL-18Rα, as a binding partner of mature IL-37, IL-18BP is expression mainly at the granular layer and basal layer in OLK tissues, and no difference between OSCC with metastasis and the normal control. We therefore propose that IL-37 achieve its extracellular functions major through its receptor IL-18Rα but not the binding partner IL-18BP and then regulate the signalling pathways downstream in the process of oral mucosa carcinogenesis.

In summary, we report a wave curve pattern of IL-37 expression with increased expression from healthy oral mucosa to oral precancerous lesions, and deceased expression from oral precancerous lesions to OSCC. A positive relationship between IL-37 expression and epithelial dysplasia in OLK has been revealed. In addition, the inhibitory effect of exogenous IL-37 expression on the expression of pro-inflammatory cytokines was also confirmed. Our results indicate IL-37 as a possible diagnostic and prognostic marker for malignant transformation of the oral mucosa. Future studies will focus on elucidating the regulatory mechanisms of IL-37-mediated immune reactions, as well as understanding its precise role in the pathology of oral mucosa cancers.

## Methods

### Patients and Tissue Microarray

A total of 70 OSCC patients were enrolled for this study. All the patients had been diagnosed with OSCC by two experienced pathologists in accordance with the Pathology and Genetics of Head and Neck Tumours of World Health Organization Classification of Tumours. Fifty-five case subjects, clinically and pathologically diagnosed as OLK were also recruited for this study. Among these, 45 patients were recruited for tissue microarray. The area for the tissue cores was selected under a microscope with a corresponding section stained with haematoxylin-eosin (HE). Tissue cores (diameter, 1.5 mm) from each donor block and were placed into a receiver paraffin block, and then cut into 4 mm sections to be used for immunohistochemical staining. Ten volunteers without any history of cancer, matched with the case subjects in gender and age distribution, were recruited as controls during the same period. All subjects enrolled were from the West China Hospital of Stomatology, Sichuan University and Guangdong Provincial Stomatological Hospital. Patients with immune diseases or histopathologically diagnosed for conditions other than OSCC or OLK were excluded.

### Ethics statement

This study was approved by the Ethics Committee both of the West China Hospital of Stomatology and the Guangdong Provincial Stomatological Hospital and was conducted in agreement with the Helsinki Declaration (Permit Number: WCHSIRB-D-2014-106). Written informed consent was obtained from all subjects and was provided by all participants at baseline and during follow-up.

### Cell lines

Normal Oral Keratinocytes (NOK), Dysplastic Oral Keratinocytes (DOK), two human OSCC cell lines (HSC-3, HSC-4), the human leukemic cell line THP-1, and the mouse mononuclear macrophage RAW264.7 cells were used in this study. NOK-SI was kindly provided by Dr. J.S. Gutkind (National Institute of Dental and Craniofacial Research). DOK was purchased from European Collection of Cell Cultures (ECACC, Salisburg, UK). HSC-3 (JCRB0623) and HSC-4 (JCRB0624) were purchased from the cell bank of the Japanese Collection of Research Bioresource (JCRB, Shinjuku, Japan). THP-1 was kindly provided by Dr. XiKun Zhou (State Key Laboratory of Biotherapy, West China Hospital, Sichuan University). RAW264.7 was purchased from the Chinese Academy of Sciences (ATCC Number: TIB-71, Beijing, China). All cells were cultured in appropriate media with 10% foetal bovine serum (PAA Laboratories, Linz, Austria) in a humidified incubator at 37 °C with 5% CO_2_.

### IHC Staining and Analysis

The tissues were embedded in paraffin, sectioned, placed on coated slides, washed with xylene to remove the paraffin, and rehydrated through serial dilutions of alcohol. The antigen was retrieved with citrate buffer (10 mM) for 3 min. Endogenous peroxidase activity was blocked with 3% H_2_O_2_ for 15 minutes and the sections were then blocked using 5% bovine serum albumin (Sigma, St. Louis, MO, USA) for 30 minutes. Slides were incubated overnight at 4 °C with anti-human IL-37b antibody (1:300 dilution; ab57187, Abcam, Cambridge, MA, USA), Human IL-18Rα antibody (8 ug/ml; MAB840, Emeryville, CA, R&D Systems), anti-IL18 binding protein antibody (1:400 dilution; ab52914, Abcam, Cambridge, MA, USA), and binding was detected with ChemMate DAKO EnVision Detection Kit (DAKO, Copenhagen, Denmark). The sections were then counterstained with haematoxylin, dehydrated and mounted^50^. Four-micrometre vertical sections of FFPE biopsies were stained with anti-human IL-37 mAb and microscopic images were acquired (original magnification ×200). The staining was assessed by three independent investigators without any knowledge of the clinico-pathological data. The following criteria were used to score the staining, first was staining intensity: 0-no detectable staining, 1-light yellow, 2-deep yellow, or 3-brown; and the second criterion was staining proportion: 0 (<5%), 1–(5–25%), 2–(25–50%), 3–(51–75%) or 4–(>75%). The sum of the two scores was considered as the final score, and it was assigned to one of four levels: 0–1 score (−), 2scores (+), 3–4 scores (++), more than 5 scores (+++). The score of staining intensity was acceptable if two or more investigators independently defined it as such^51^,^52^.

### Recombinant human IL-37

Recombinant human IL-1F7b (rhIL-37b) was purchased from R&D systems (7585-IL-025, 25 ug).

### Real-time Quantitative PCR (Q-PCR)

Transfected THP-1 cells were differentiated into macrophages by treatment with PMA (50 ng/ml). After 24 hours, all the cells were counted and plated; undifferentiated cells were resuspended immediately, whereas differentiated cells were incubated. The cells were then cultured overnight with serum-free medium. After 24 hours of incubation with 50 ng/ml of rhIL-37b and 1 ug/ml of LPS, total RNA was isolated from the cultured cells using Trizol reagent (Invitrogen, Carlsbad, CA, USA) and reversed transcribed using PrimeScript RT reagent kit (TaKaRa, Bio, Otsu, Japan). Q-PCR was performed using the SYBR Premix Ex Taq II kit (TaKaRa, Bio, Otsu, Japan) on a 7300 real-time PCR system (Applied Biosystems, Invitrogen, Carlsbad, CA, USA) with gene-specific primers. Endogenous β-actin expression was used as an internal control. Primers were purchased from TSINGKE Biological technology and are listed in Supplementary Table 1.

### Western blot

All cells were subjected to protein extraction and western blot analysis with a primary antibody specific for pro-IL-37b (mouse monoclonal, 1:2000, Proteintech, Wuhan, China) and mat-IL-37b (2 ug/ml; AF840, Emeryville, CA, R&D Systems). GAPDH antibody (mouse monoclonal, 1:5000, Sigma, St. Louis, MO) was used as an internal control.

### Statistical analysis

The data were analysed by using the Statistical Package for Social Science (SPSS) version 17.0 for Windows. The rank sum test was used to compare the differences in IHC staining of tissues from different groups. For all statistical analyses, a P value of less than 0.05 was considered as significant.

## Additional Information

**How to cite this article**: Lin, L. *et al.* Interleukin-37 expression and its potential role in oral leukoplakia and oral squamous cell carcinoma. *Sci. Rep.* **6**, 26757; doi: 10.1038/srep26757 (2016).

## Supplementary Material

Supplementary Information

## Figures and Tables

**Table 1:** Expression of IL-37 in OLK, OSCC and normal controls.

Clinicopathology	Total n	Expression of IL-37			
		Negative n	Weak n	Strong n	P value
		(percentage)	(percentage)	(percentage)	
Normal control	10	4 (40%)	5 (50%)	1 (10%)	
Oral leukoplakia	10	0 (0%)	1 (10%)	9 (90%)	0.001
Oral squamous cell carcinoma	10	0 (0%)	2 (20%)	8 (80%)	0.001

**Table 2:** Association of IL-37 expression in OLK with patients’ clinicopathological characteristics (n = 45).

	Total n	Negative n	Weak n	Strong n	P value	
		(percentage)	(percentage)	(percentage)		
Gender						
Female	27	11 (40.7%)	13 (48.1%)	3 (10.7%)	0.876	
Male	18	5 (27.8%)	12 (66.7%)	1 (5.6%)		
Age						
<60	32	12 (37.5%)	17 (53.1%)	3 (9.4%)	0.874	
≥60	13	4 (30.8%)	8 (61.5%)	1 (7.7%)		
Smoking status						
Smoker	22	6 (27.3%)	14 (63.6%)	2 (9.1%)	0.464	
Nonsmoker	23	10 (43.5%)	11 (47.8%)	2 (8.7%)		
Epithelial dysplasia						
Without epithelial dysplasia	29	15 (51.7%)	14 (48.3%)	0 (0%)	0.001	
Mild epithelial dysplasia	12	1 (8.3%)	9 (75%)	2 (16.7%)		
Moderate epithelial dysplasia	4	0 (0%)	2 (50%)	2 (50%)		

**Table 3:** Association of IL-37 expression in OSCC with patients’ clinicopathological characteristics (n = 60).

	Total n	Negative n	Weak n	Strong n	P value	
		(percentage)	(percentage)	(percentage)		
Gender						
Female	44	8 (18.2%)	20 (45.4%)	16 (36.4%)	0.468	
Male	16	2 (12.5%)	5 (31.3%)	10 (56.2%)		
Age						
<60	31	6 (19.4%)	11 (35.5%)	14 (45.2%)	0.349	
≥60	29	4 (13.8%)	14 (48.3%)	11 (37.9%)		
Smoking status						
Smoker	35	5 (14.3%)	16 (45.7%)	14 (40.0%)	0.636	
Nonsmoker	25	5 (20.0%)	9 (36.0%)	11 (44.0%)		
Differentiation						
Well	34	5 (14.7%)	14 (41.2%)	14 (44.1%)	0.350	
Medium/Poor	26	5 (19.2%)	11 (42.3%)	10 (38.5%)		
Tumor stage						
T1–T2	42	8 (19.0%)	20 (47.6%)	14 (33.3%)	0.365	
T3–T4	18	2 (11.1%)	5 (27.8%)	11 (61.1%)		
Nodal stage						
N0	30	3 (10.0%)	12 (40.0%)	15 (50.0%)	0.006	
N1–Nx	30	7 (23.3%)	13 (43.3%)	12 (33.3%)		
Clinical TNM stage						
I	9	0 (0.0%)	4 (44.4%)	5 (55.6%)	0.082	
II	12	3 (25.0%)	5 (41.7%)	4 (33.3%)		
III	31	5 (16.1%)	15 (48.4%)	11 (35.5%)		
IV	8	2 (25.0%)	1 (12.5%)	5 (62.5%)		
