# Supplementary material for: Interleukin-37 expression and its potential role in oral leukoplakia and oral squamous cell carcinoma
Source: Sci Rep. 2016 May 26;6:26757. doi: 10.1038/srep26757 (PMC4880905; doi:10.1038/srep26757)
Supplement: Supplementary Information [file srep26757-s1.doc]

**Interleukin-37 expression and its potential role in oral leukoplakia and oral squamous cell carcinoma**

Lin Lin^1, 2, #^, Jiayi Wang^1, 3, #^, Dongjuan Liu^1, #^, Sai Liu^1^, Hao Xu^1^, Ning Ji^1^, Min Zhou^1^, Xin Zeng^1^, Dunfang Zhang^1^, Jing Li^1, *^, Qianming Chen^1, *^

**Email address**

Lin Lin: [409083171@qq.com](mailto:409083171@qq.com) Jiayi Wang: jiayimail@163.com

Dongjuan Liu: djliu1005@163.com Sai Liu: [liusaisay@163.com](mailto:liusaisay@163.com)

Hao Xu: xhstatis@hotmail.com Ning Ji: [32405973@qq.com](mailto:32405973@qq.com)

Min Zhou: 1551779457@ qq.com Xin Zeng: [zengxin22@163.com](mailto:zengxin22@163.com)

Dunfang Zhang: 330741854@qq.com

Jing Li*: [lijing19840108@126.com](mailto:lijing19840108@126.com) Qianming Chen*: qmchen@scu.edu.cn

**Department**

^1^ State Key Laboratory of Oral Diseases, West China Hospital of Stomatology, Sichuan University, Chengdu, Sichuan, China; ^2^ Nanjing Stomatological Hospital, Medical School of Nanjing University, Nanjing, Jiangsu, China; ^3^ Department of Oral Radiology, West China College of Stomatology, Sichuan University, Chengdu, Sichuan, China.

^#^ Authors contributed equally to this work.

**Corresponding author**

Jing Li*, Qianming Chen*, State Key Laboratory of Oral Diseases, West China Hospital of Stomatology, Sichuan University, No. 14, Sec. 3, Renminnan Road, Chengdu, Sichuan, 610041, China, 610041, +86 28 85503484; Tax: +86 28 85503484; E-mail: lijing19840108@126.com (J. L.), qmchen@scu.edu.cn (Q.C.).

**Supplementary Figures**


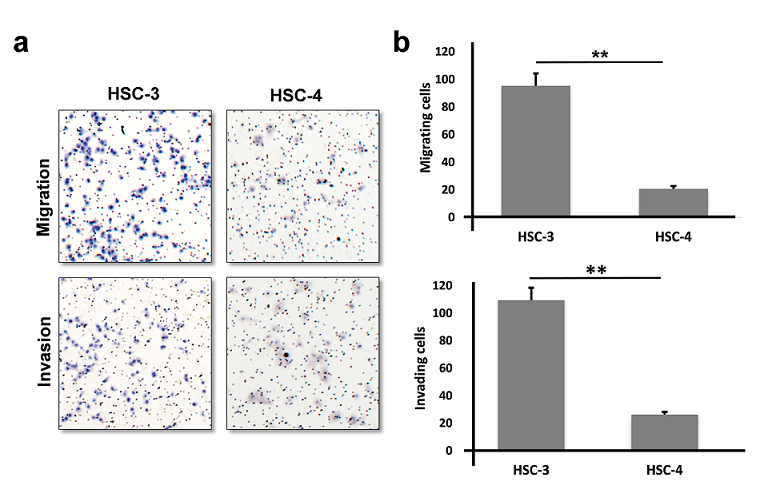


**Supplementary Figure 1.** HSC-3 cells have more invasion and migration capacity than HSC-4 cells. A) Both 5*10^4^ cells of HSC-3 and HSC-4 were re-suspended with serumfree Dulbecco’s modified eagle medium (DMEM, Gibco, Invitrogen) and then seeded in a cell culture insert (pore size 8 mm, BD Biosciences) for 24-well plate in triplicates. DMEM containing 10% fetal bovine serum was added to the lower chamber. 14 hours later, cells in the upper chamber were wiped using a cotton stick and cells migrated to the other side of the chamber were fixed with 70% methanol and stained by crystal violet. Procedures of invasion assay were similar to migration assay except for that BioCoat Matrigel Invasion Chambers (BD Biosciences) were used instead of uncoated inserts, and cell number and incubation time were doubled. B) The relative numbers of HSC-4 cells migrated or invaded to the lower surface of the matrigel-coated cell culture insert. All assays were carried out three times in triplicates (*P<0.05, **P<0.01).


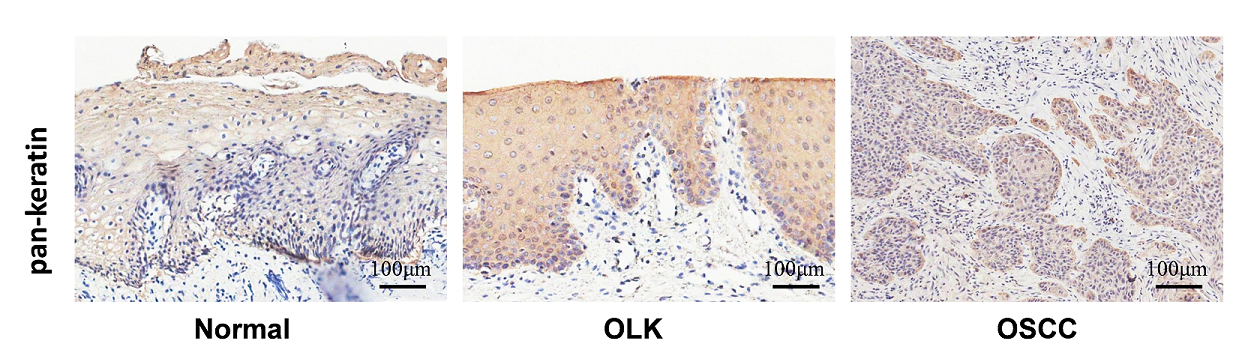


**Supplementary Figure 2.** The expression of pan-keratin in different stages of carcinogenesis tissues. Moderate cytomembrane and cytoplasmic staining of keratin was detected in all of the NC and OSCC samples, whereas OLK samples showed positive staining in all of the epithelial, especially in the keratin layer.

**Supplementary Tables**

Supplementary Table 1. Primer design for real-time quantitative PCR

| **Gene** | **Primer sequences** |
| --- | --- |
| IL-6 | Forward: 3’- ACTCACCTCTTCAGAACGAATTG -5’ |
|  | Reverse: 5’- CCATCTTTGGAAGGTTCAGGTTG -3’ |
| TNF-α | Forward: 3’- CCTCTCTCTAATCAGCCCTCTG -5’ |
|  | Reverse: 5’- GAGGACCTGGGAGTAGATGAG -3’ |
| IL-1β | Forward: 3’- TTCGACACATGGGATAACGAGG -5’ |
|  | Reverse: 5’- TTTTTGCTGTGAGTCCCGGAG -3’ |
| IL-10 | Forward: 3’- GACTTTAAGGGTTACCTGGGTTG -5’ |
|  | Reverse: 5’- TCACATGCGCCTTGATGTCTG -3’ |
| IL-37 | Forward: 3’- CAAGCCTCCCCACCATGAATTT -5’ |
|  | Reverse: 5’- GCAAAGAAGATCTCTGGGCGT -3’ |

**Supplementary Table 2. The statistical analysis of IL-18Rα and IL-18BP in NC, OLK and OSCC tissues.**
